# A novel method of inducing endogenous pupil oscillations to detect patients with unilateral optic neuritis

**DOI:** 10.1371/journal.pone.0201730

**Published:** 2018-08-22

**Authors:** Cedric Lamirel, Suzon Ajasse, Antoine Moulignier, Laurence Salomon, Romain Deschamps, Antoine Gueguen, Catherine Vignal, Isabelle Cochereau, Jean Lorenceau

**Affiliations:** 1 Departement d’ophtalmologie, Fondation Ophtalmologique Adolphe de Rothschild, Paris, France; 2 Service d’ophtalmologie, Hôpital Bichat- Claude Bernard, Paris, France; 3 Institut de la Vision, UPMC, Inserm-CNRS, Paris, France; 4 Service de neurologie, Fondation Ophtalmologique Adolphe de Rothschild, Paris, France; 5 Unité de recherche clinique, Fondation Ophtalmologique Adolphe de Rothschild, Paris, France; 6 Université Paris Diderot, Paris, France; Bascom Palmer Eye Institute, UNITED STATES

## Abstract

**Purpose:**

To use and test a new method of inducing endogenously generated pupillary oscillations (POs) in patients with unilateral optic neuritis (ON), to describe a signal analysis approach quantifying pupil activity and to evaluate the extent to which POs permit to discriminate patients from control participants.

**Method:**

Pupil size was recorded with an eye-tracker and converted in real time to modulate the luminance of a stimulus (a 20° disk) presented in front of participants. With this biofeedback setting, an increasing pupil size transforms into a high luminance, entraining a pupil constriction that in turn decreases the stimulus luminance, and so on, resulting in endogenously generated POs. POs were recorded for 30 seconds in the affected eye, in the fellow eye and in binocular conditions with 22 patients having a history of unilateral ON within a period of 5 years, and with 22 control participants. Different signal analysis methods were used to quantify the power and frequency of POs.

**Results:**

On average, pupil size oscillated at around 1 Hz. The amplitude of POs appears not to be a reliable marker of ON. In contrast, the frequency of POs was significantly lower, and was more variable over time, in the patients’ affected eye, as compared to their fellow eye and to the binocular condition. No such differences were found in control participants. Receiver operating characteristic analyses based on the frequency and the variability of POs to classify patients and control participants gave an area under the curve of 0.82, a sensitivity of 82% (95%CI: 60%-95%) and a specificity of 77% (95%CI: 55%-92%).

**Conclusions:**

The new method used to induce POs allowed characterizing the visual afferent pathway defect in ON patients with encouraging accuracy. The method was fast, easy to use, only requiring that participants look ahead, and allows testing many stimulus parameters (e.g. color, stimulus location, size, etc).

## Introduction

The pupil regulating the quantity of light entering the eyes is controlled by dedicated circuits. Pupil constriction is mediated the parasympathetic pathway which originates from melanopsin-containing, intrinsically photosensitive, retinal ganglion cells (ipRGCs), a special class of RGC that do not project to the LGN but to the pretectal olivary nucleus (PON). Neurons from the PON project to the Edinger–Westphal nucleus (EW) that then innervate the ciliary ganglions driving the sphincter muscle that constricts the iris. [1–5] Pupil dilation relies on a 3-neurons sympathetic circuit originating in the hypothalamus, then projecting to the superior cervical ganglion, after descending in the spinal cord and climbing along the internal carotid artery and the ophthalmic artery and ending in the pupillary dilator muscle.[1,2,6] Pupil dilation arises from both an excitation of the sympathetic nerve and a central inhibition of the parasympathetic pathway.[2,6–9]

Pupillary responses have long been studied to determine the extent to which they provide reliable markers of optic neuropathies and retinopathies.[1] Abnormalities of pupil reactivity to light stimuli include alterations of the latency and amplitude of the pupillary light reflex (PLR) elicited by brief stimuli or of the amplitude and phase-lag responses to periodic changes in light intensity. These alterations can occur in many neuropathies, including glaucoma or optic neuritis (ON)[10–16] or retinopathies (age related macular disease).[17]

**Fig 1:**
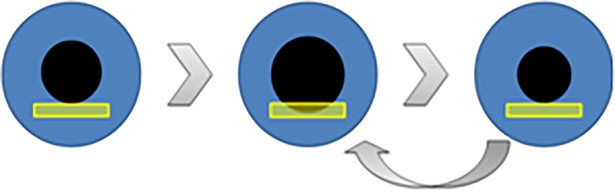
Pupil cycle time elicited by positioning the beam of a slit lamp at the inferior margin of the pupil. With this method, the pupil cycle time is derived from the number of cycles measured using a stop watch during a fixed period.

Another approach developed by Miller and Thomson investigated the characteristics of the pupil cycle time (PCT) to seek whether it provides a quantitative marker of ON.[18,19] In their study, periodic cycles of dilation and constriction are induced by illuminating the pupil margin using a thin beam of a slit-lamp, placed in such a way that pupil size “controls” the amount of light entering the eye through biofeedback: the beam of light first entrains a pupil constriction, such that the beam light then falls outside the pupil, not entering the eye; the so induced decreased retinal illumination in turn entrains a pupil dilation, such that the beam light enters the eye again, eliciting a constriction, and so on (Fig 1).

Measuring the peak-to-peak time between oscillations revealed that the PCT is stable, repeatable and is significantly longer for patients with ON. Although Miller and Thompson[18,19] and follow-up studies[20] showed that PCT measures are simple and indeed objectively and quantitatively reflect pupillary defects linked to dysfunction of the afferent part of the PLR, their method suffers from some limitations:

- PCT measures require a good practice to master the slit-lamp positioning, and any eye or head movement of the participant may disrupt the pupil cycling,
- The slit-lamp stimulus can only be easily applied to the iris margin; hence, it cannot be used to test specific foveal or other retinal regions, or to investigate different stimulus characteristics (for instance stimulus color, size, structure, position or extent),
- Recording pupil activity allows quantitative off-line analyses to characterize the time and variability of pupil oscillations. The development of graphics displays and of video based eye-tracking devices open the way to a thorough characterization of pupil activity over time. Recording pupil oscillations with an eye-tracker permit off-line analyses to compute many variables (e.g. frequency, amplitude, stability of the oscillations), and to remove blinks or saccade that could alter pupil activity. Importantly, it also permits simultaneous measures both pupils.

In the clinic, pupil reactivity to light (PLR) and the relative afferent pupillary reflex (RAPD) are used to detect lesions of the optic nerve with standardized available devices. The RAPD allows detecting asymmetrical amplitude of pupil constriction in response to alternating light stimulation of each eye, only allowing intra-individual assessment of pupil reactivity, and being useless whenever both optic nerves are symmetrically affected. Here, we present a simple and novel method to induce endogenous pupil oscillations (POs) that avoids some of the above-mentioned caveats, and allows comparing the two eyes of the patients and comparison between patients and control subjects.

As the size of the pupil recorded by the eye-tracker varies as a function of the eye-camera settings (angle relative to, or distance to, the eyes) and as the amplitude of pupil responses is known to be influenced by cognitive factors[8,9]; we focus our analyses on the temporal characteristics of POs.

In the following, we first describe the principle of our method; we then present the results obtained on a population of 22 patients with a history of unilateral ON, and 22 matched control subjects. Using a signal analysis approach to compute pupil data, we show that the power of POs is not as reliable as the frequency of pupil oscillation to detect defects in the afferent pathway of the affected eye of patient with ON, and that this variable is a behavioral biomarker of ON.

## Method

### General principle

**Fig 2:**
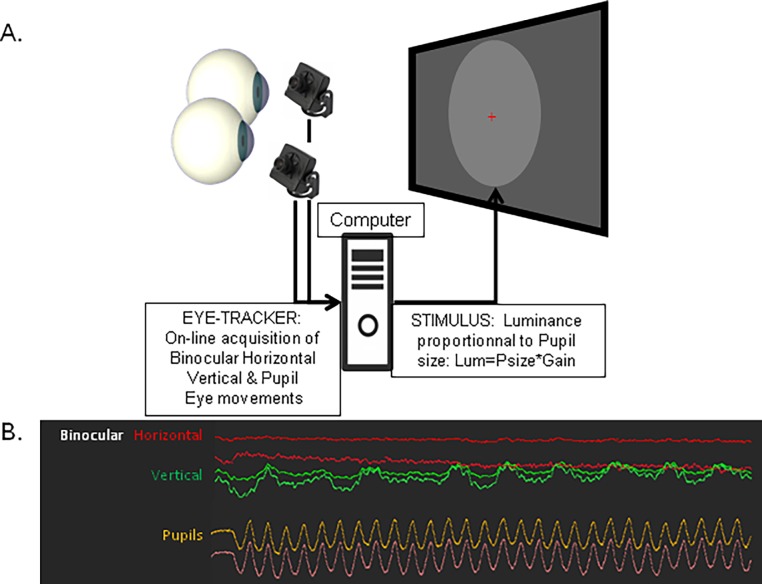
Device used in the study. A. Eyes positions and pupil data are recorded binocularly and stored for off-line analyses. Pupil data are converted on-line into a luminance level, (Luminance = Pupil Size* Gain) to modulate a 20° disk stimulus. In this configuration, the pupil enters an oscillatory pattern, whose frequency was used to characterize pupillary defects. B. Example of 30 seconds of recording showing eye positions (red/green traces) and binocular pupil oscillations (yellow and orange). Note that oscillations of vertical eye position reflect respiratory artifacts.

The method, illustrated in Fig 2, implies recording pupil size with an eye-tracker. Each recorded sample of eye data is sent to a computer that transforms pupil size into a variable that controls the luminance of a stimulus presented on a computer screen in front of the subject. To ensure that the stimulus luminance remains in a suitable range, pupil size delivered by the eye-tracker is transformed in real-time into luminance by dividing pupil size with a constant parameter. This value, or Gain, is assessed for each participant prior to the main experiment by measuring the maximum size of the pupil in response to a dark screen. The Gain is then set so as to maintain the stimulus luminance in a range not exceeding the graphics display capabilities (in the 0–255 range). More precisely, pupil size delivered by the eye-tracker is in arbitrary units with values ranging between 3000 and 15000. To convert these values into luminance levels ranging from 0 to 255, we divided pupil size by this Gain. As the gain value depends on the intrinsic pupil size of a participant, and on the distance between the camera and the eyes, it had to be estimated for each participant at the beginning of a session, in order to maximize the amplitude of pupil oscillations while keeping luminance in a suitable range.

In this situation, an increasing pupil size induces an increase of the stimulus luminance, which in turn entrains a constriction of the pupil, hence producing a luminance decrease, and so on. As pupil size and display luminance are coupled in real-time through biofeedback (with a delay not exceeding the Inter Frame Interval, IFI, i.e. 13.33 msec. with a 75 Hz display, which is negligible when compared to pupil dynamics), the pupil enters a sustained oscillatory activity characterized by its amplitude and its frequency. As oscillatory patterns are best analyzed in the frequency domain using a Fast Fourier Transform (FFT) or other signal analysis methods, we refer in the following to Pupil Oscillation Frequency (POF). In line with previous authors[18], we propose that these oscillations reflect the time constant of the transmission of signals from ipRGCs to the iris sphincter, including the transmission time through the optic nerve. Note that if the transmission delay through the optic nerve was instantaneous (or very short), pupil oscillations would not occur as the system would quickly converge toward stable pupil/luminance equilibrium. This is not the case as the frequency of oscillation observed with this setting is around 1 Hz. As the fibers of the optic nerves are, at least in part, demyelinated in ON patients, we expected that they exhibit a slowing down the propagation speed of neural activity, and hence a lower frequency of pupil oscillations.

### Participants

Twenty-two patients (mean age: 37 years, range 24–60) from a neurological clinic with a history of unilateral demyelinating ON within a 5 year-period participated in the study (see Table 1 for a description of the patient group). A group of 22 healthy participants matched in age and sex was also recruited after a visual examination with a trained orthoptist assessing their past medical and ophthalmologic history, visual acuity, absence of relative afferent pupillary defect, non-contact intraocular pressure, central corneal thickness, fundus photography. All the charts were reviewed by a neuro-ophthalmologist and glaucoma specialist (CL). None of the participants presented any anisocoria that could reflect lesions of the efferent part of the PLR. In the following, the affected eye was the eye with a history of demyelinating ON and the fellow eye was the unaffected eye. For the healthy participants, the affected eye was the eye on the same side as the affected eye of their matched patient, and the fellow eye was the eye on the same site of the unaffected eye of their matched patient.

**Table 1:** Description of patient’s history and pathology.

	Sexe	Age	Affected Eye	Time since ON	Visual acuity OD (snellen equivalent)	Visual acuity OS (snellen equivalent)	MS status according to 2005 revised MacDonald criteria
Patient 1	M	24	OD	10 months	20/20	20/20	RR MS (2010)
Patient 2	F	37	OS	6 months	20/25	20/20	RR MS (2010)
Patient 3	F	29	OD	11 days	CF	20/20	CIS
Patient 4	M	40	OD	10 months	20/30	20/20	CIS
Patient 5	F	26	OS	12 days	20/20	20/20	RR MS (2009)
Patient 6	F	55	OS	3 months	20/20	20/25	RR MS (2003)
Patient 7	M	38	OD	9 months	20/20	20/20	RR MS (2012)
Patient 8	F	44	OS	57 months	20/20	20/20	RR MS (2010)
Patient 9	F	26	OS	4 months	20/20	CF	CIS
Patient 10	F	31	OS	5 months	20/20	20/20	CIS
Patient 11	M	31	OD	5 months	20/20	20/20	RR MS (2012)
Patient 12	F	33	OD	1 months	20/200	20/20	CIS
Patient 13	M	51	OD	9 months	20/50	20/20	SP MS (1993)
Patient 14	F	27	OS	16 months	20/20	20/20	RR MS (2012)
Patient 15	F	38	OS	3 months	20/20	20/20	RR MS (2007)
Patient 16	M	44	OS	36 months	20/30	20/20	RR MS (2010)
Patient 17	M	60	OD	37 months	20/20	20/20	PP MS (2009)
Patient 18	F	34	OD	60 months	20/20	20/20	RR MS (2008)
Patient 19	F	24	OS	58 months	20/20	20/20	RR MS (2011)
Patient 20	F	37	OD	9 days	20/20	20/20	RR MS (2013)
Patient 21	F	31	OD	10 months	20/20	20/20	CIS
Patient 22	F	48	OD	2 months	20/25	20/20	RR MS (2012)

MS: Multiple Sclerosis; CIS: Clinically Isolated Syndrome; RR MS: Relapsing-Remitting MS; SP MS: Secondary Progressive MS; PP MS: Primary Progressive MS.

The choice of studying POs in patients with unilateral ON permits to tests our method both for within and between groups (patients versus controls) differences. We expect that in the patient group, the POs of the affected eye will be lower than in their fellow eye, and that the affected eye of the patients will be lower than the paired eye of the control participants.

This study follows the tenets of the Declaration of Helsinki. Informed consent was obtained from the participants after explanation of the nature and possible consequences of the study which had been approved by the ethic committee of the “CPP Ile-de-France VI, Groupe hospitalier Pitié-Salpêtrière”. This study was registered at http://www.clinicaltrials.gov (identifier: NCT02004054 and NCT02894281)

### Stimuli

The stimuli were displayed on a 22 inch monitor (IIYAMA, 30.48 cm vertical viewable screen size, screen resolution of 1024 x 768 pixels, 75Hz frame rate, 8 bpp) viewed from a distance of 67cm. The stimulus consisted of a 20° diameter disk displayed on an otherwise black screen. Eye data were sampled at 250 Hz (Inter Frame Interval = 4 ms) by an Eye-Link II eye-tracker (SR Research Ldt). A dedicated custom software (JEDA) was used for coupling pupil size and luminance, and for recording the eye-data, at the screen refresh rate (75 Hz, IFI = 13.33 ms). To determine the maximum and minimum pupil size of each participant, every trial started with a pupil calibration procedure: 3 seconds with the lowest luminance (0.003 cd/m^2^), 3 seconds with maximum luminance (90 cd/m^2^), 3 seconds with a medium luminance (45 cd/m^2^); we then used these values to z-normalized the pupil size.

Pupil oscillations were then induced in the affected eye, in the fellow eye, and in binocular condition. Two runs were performed for each condition, resulting in 6 recordings per participant.

Data collected with white, red, green, and blue luminance modulations indicated that the green stimulus was more efficient than the other colors. The origins of these differences between colors are unclear. One possible account is that the Gain value may have been inappropriate to induce reliable POs with the red and blue colors, whose intensities are lower. Another possibility is that white, red, and blue stimuli induced more photophobia, more blinks and more saccades resulting in decreased signal/noise ratio of the pupil signal compared to the green stimulus.[21] For the sake of simplicity, we choose to only report the results obtained with a green stimulus here.

### Task

Participants removed their corrective lens, if any, to avoid possible artifacts that could perturb eye-recordings. They then dark adapted during 5 minutes to the dark testing room. A 9 points eye-calibration was performed before running the different experimental conditions, each starting with the recording of the PLR for 3 luminance levels (dark, middle and maximum luminance), followed by the induction of POs. Participants were instructed to maintain fixation on a central fixation point and to avoid blinking as much as possible. Each recording epoch (30 seconds), was separated from the next by a period of rest (~30 seconds).

### Data analyses

Ad-hoc programs were developed using Matlab (The Maths Work, Inc.) to analyze the pupil data. Blinks were automatically detected and replaced by a linear interpolation between the start and end of each blink. Each raw data file was visually checked, and missed blinks or artifacts were manually replaced by a linear interpolation. We then computed a z-normalization of each recording of each participant. As pupil oscillated smoothly over time, we computed the fundamental frequency of endogenous pupil oscillations with several methods: Fast Fourier Transform (FFT), autocorrelation and FFT of the auto-correlograms, and time-frequency analysis (Time Frequency maps using a Morlet complex wavelet m = 10). As the power spectrum follows a 1/f function with more power in the low frequency range that could conceal the PO frequency (POF), the power spectrum was flattened by multiplying the power at each frequency by the corresponding squared frequency. Based on previous results[18–20], we concentrated our analyses on the frequencies with maximum power between 0.5 and 2 Hz.

**Fig 3:**
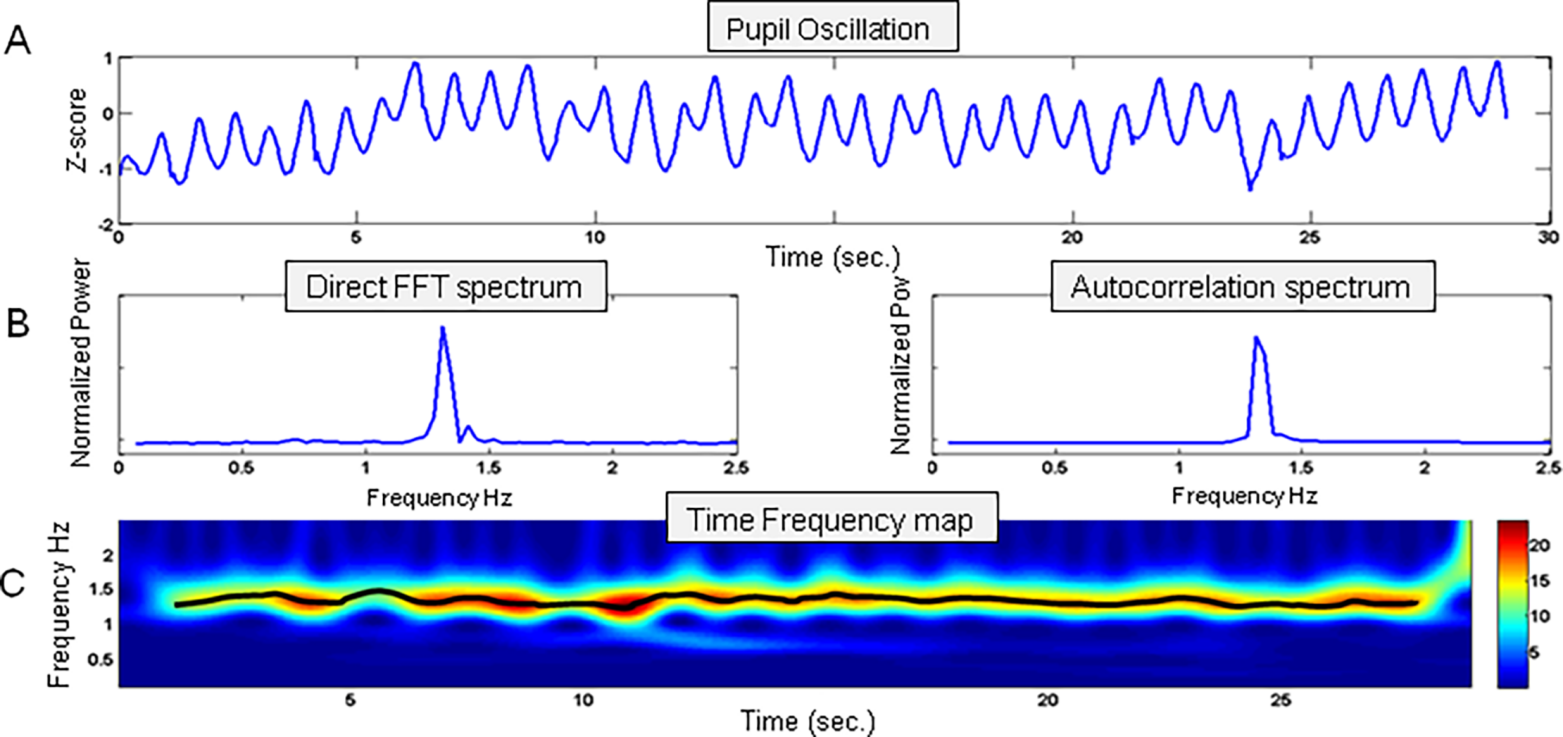
Example of a single trial analyses. A) Z-normalized pupil size recorded for 30 seconds. B) Power spectrum and auto-correlogram spectrum of the z-normalized pupil data. C) Time frequency map showing the frequency power as a function of time: the power at each time step is color-coded; with red indicating a high power and blue a low power. The black line represents the maximum power frequency at each time step (black curve). The mean and variance of this maximum frequency distribution was used to characterize pupil dynamics.

Fig 3 presents an example of the performed analyses: the upper panel shows z-normalized pupil size recorded over 30 seconds, the middle panels shows the power spectrum and autocorrelation, and the lower panel shows the time-frequency plot.

**Fig 4:**
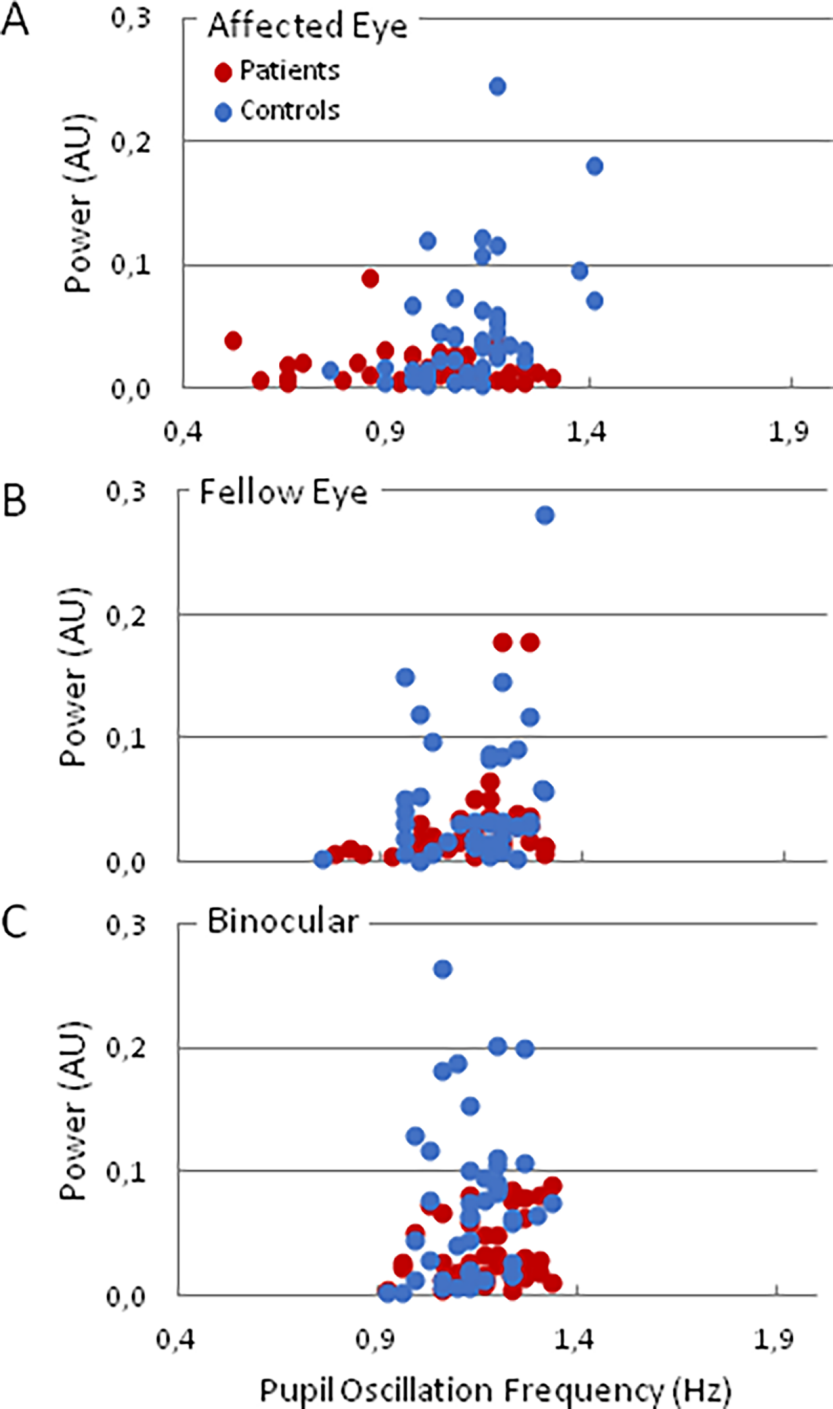
Power and frequency of POs obtained for the affected and fellow eyes, and in binocular conditions. Each dot represents the result of one participant. Note that the power of POs depends little on the condition (affected, fellow and binocular conditions), while the POF shows larger differences between conditions.

The pupil size measured by the eye-tracker (and thus the amplitude of the POs) depends on the eye-camera distance and orientation, which were adapted differently for each participant, introducing a source of variability between subjects. The eye-camera distance and orientation were also different between the right and left eyes of the participants introducing a source of variability between the two eyes of the same subject. Finally, between each trial, the participants could rest and remove the head from the chin-rest that could result in small changes in the eye-camera distance and orientation between two trials introducing a source of variability within the same eye. Altogether these sources of variability could overweight the difference of POF amplitude between affected eye and normal eye measured by the eye-tracker. Indeed, the variation coefficient of the PO power was about 10 times larger than that of the frequency of PO. In addition, recent studies found that the amplitude of the PLR, but not its latency, was modulated by cognitive factors (i.e. attention),[9] suggesting that the temporal characteristics of pupil responses are better suited to identify deficits of the visual afferent pathways. As a matter of fact, we found that power and the frequency of POs were not strongly correlated (Fig 4).

These findings suggest that the power of POs may not provide a reliable marker of ON, as was confirmed by statistical analyses, not detailed here. Accordingly, we concentrated our analyses on PO frequency in the following.

Although the different methods gave similar results (S1 Fig), the time-frequency method (TF) method provided more stable results, offered a better understanding of pupil activity over time, and further allowed to compute the POF variance over time. We therefore focus on the results obtained with this method in the following.

## Results

In the ON group, 16 trials were removed from the analyses, either because the screen luminance rose to ceiling for more than half the trial duration, indicating that the gain, G, had not been correctly set for these particular cases, or because of too many saccades and blinks. A total of 116 trials from the group of patients with ON were thus retained for the analysis. All the 132 trials from the control group were retained for the analysis.

**Fig 5:**
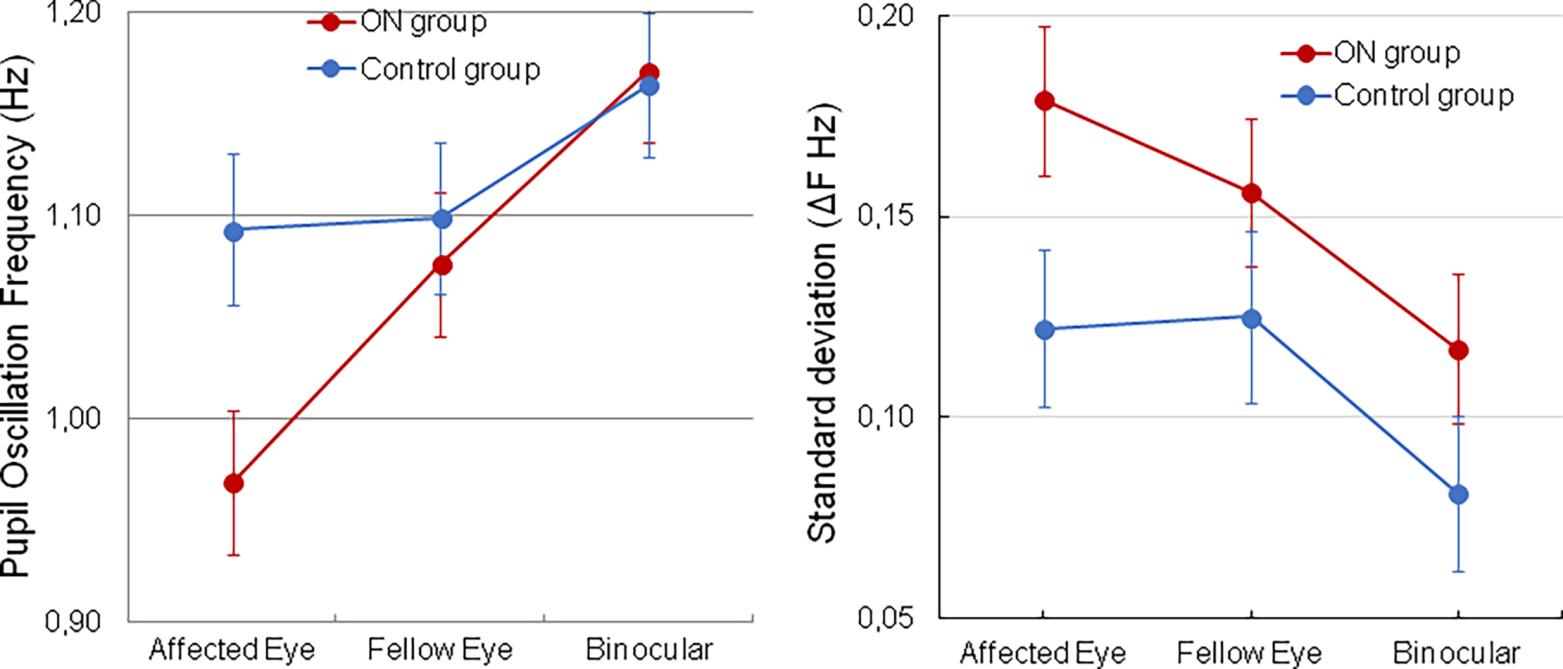
Pupil oscillation results. A. Pupil Oscillation Frequency averaged across participants for the ON (red) and control group (blue). Thin lines represent 95% confidence interval. B. Standard deviations of the POF with maximum power in the 0.5–2 Hz range, computed from the time-frequency maps.

A statistical analysis (ANOVA, S1 Table) using Group (ON, Control) and Eye (affected, fellow, binocular) as main factors, indicated that the PO peak frequency between 0.5 Hz and 2 Hz was significantly lower in the affected eye of ON group compared to fellow eye of ON group (within group comparison: Tukey’s HSD test p = 10^−3^,Fig 5A) and to both eye of the control group (inter-group comparison: Tukey’s HSD test p<10^−4^, Fig 5A). No difference was found between the fellow eye of the ON group compared to the fellow eye of the control group (Tukey’s HSD test p = 0.96). Moreover, the POF was higher in the binocular condition as compared to the fellow eye in both group (Tukey’s HSD test <10^−4^). The same differences between the affected eye, the fellow eye and in binocular conditions were found with the direct FFT and the autocorrelation methods used to identify the peak frequency; however, the importance of these differences slightly differ with the method (see S1 Fig).

The POF variability (the standard deviation, SD, of the maximum of the POF computed across time in the TF analysis, see Fig 3) was analyzed with an ANOVA using Group and Eye as main factors (see S2 Table). This analysis revealed that POF variability was overall more important in the ON group (p<10^−5^) and lower in binocular condition (Tukey’s HSD test p = 10^−4^) as shown in Fig 5B.

**Fig 6:**
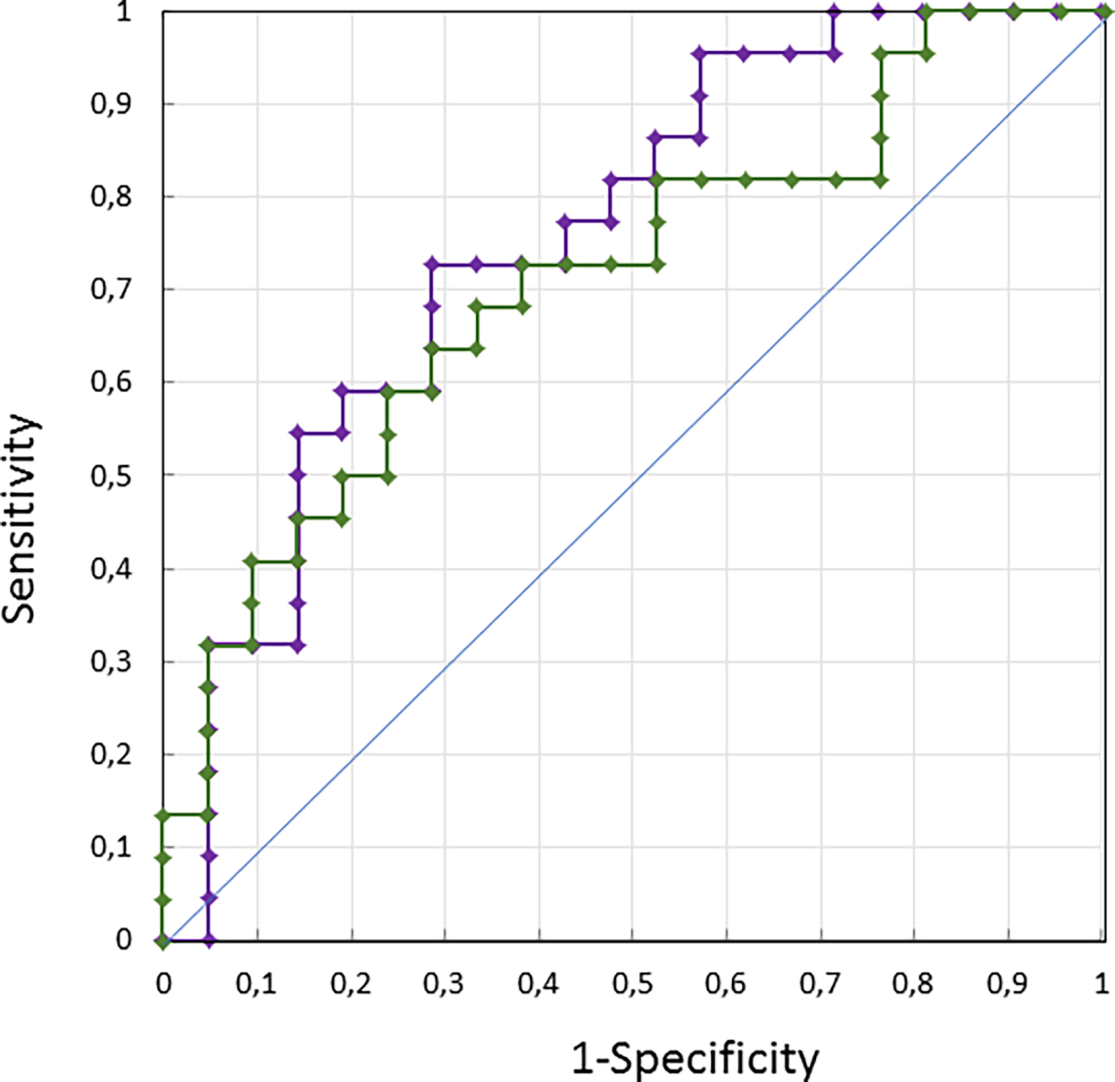
Receiver operating characteristic curves using the affected and fellow eyes of the ON patients group. ROC curves are computed with POFs (green dots) and Mixed Index (purple dots).

**Fig 7:**
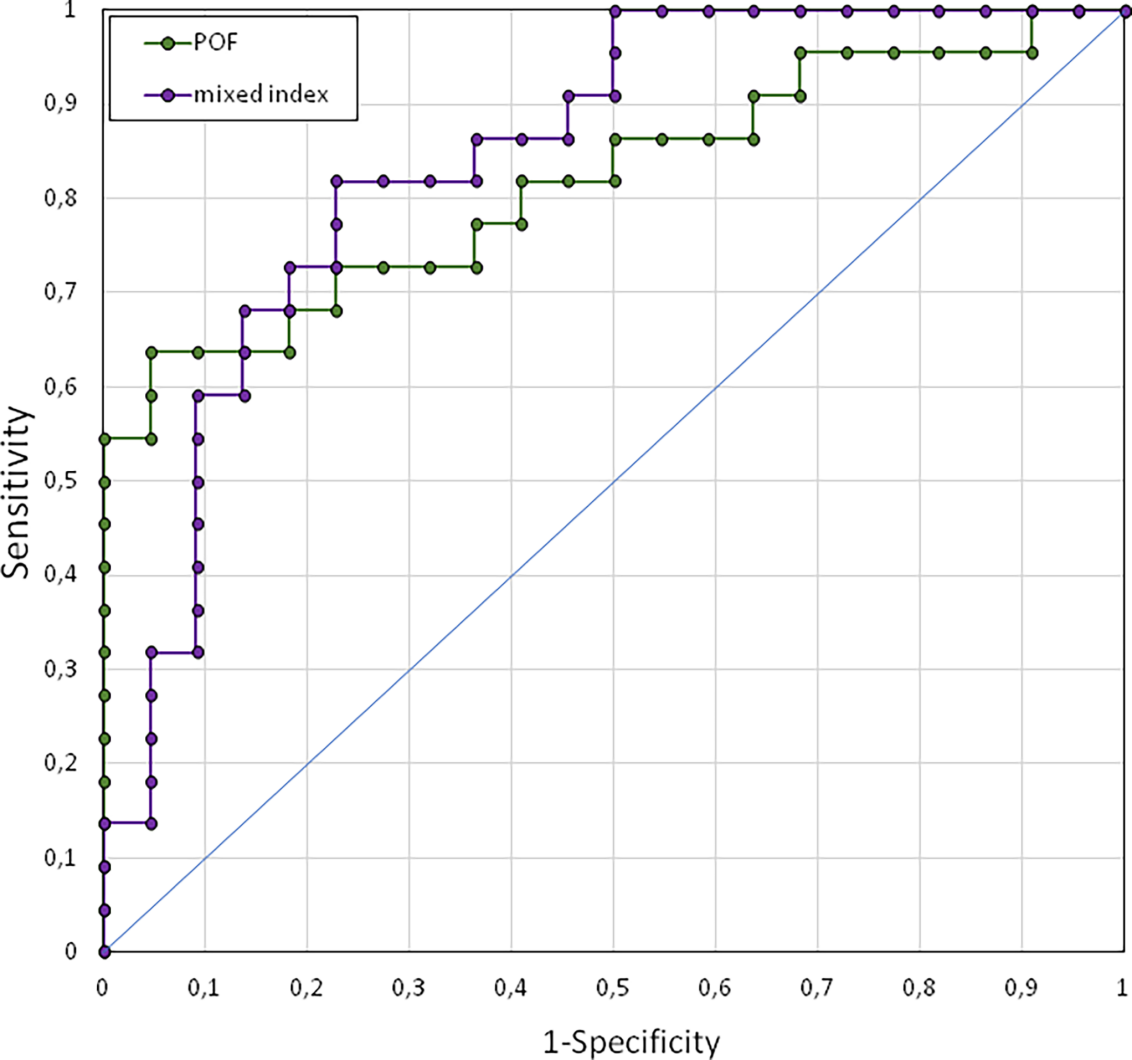
Receiver operating characteristic curves using the affected eyes of the ON patient group and the paired “affected eyes” of the Control group. ROC curves are computed with POFs (green dots) and Mixed Index (purple dots).

To determine the accuracy with which the POF can be used to classify the affected and fellow eyes of patients with unilateral ON, or the affected eye of patients with the paired eye of control subjects, we computed receiver operating characteristic (ROC) curves using individual results obtained with the TF method. We first describe the results obtained with the affected and fellow eyes of ON patients (Fig 6), before presenting the results obtained with the affected eye of ON patients and the “paired” eye of control subjects (Fig 7). We performed these ROC analyses using two indices: the value of the POF alone, or a mixed index corresponding to the ratio of the POF to its variability (POF/SD of POF). This mixed index takes advantage of the finding that POF variability is larger in the affected, as compared to the fellow eye of patients and in patients as compared to control participants. Combining POF and its variability is expected to maximize the sensitivity of our method.

For the patients (Fig 6), and using solely the POF, we found an Area Under the Curve (AUC) of 0.76, a sensitivity of 73% (95%CI: 50%-89%) and a specificity of 71% (95%CI: 48%-89%) with a threshold of 1.02 Hz. Of the 22 patients 16/22 affected eyes, and 15/21 fellow eyes, were correctly classified. Using a mixed index did not increase these values (AUC 0.71, sensitivity 68%; 95%CI: 45%-86% and specificity 67%; 95%CI: 43%-85%); 15/22 affected eyes and 14/21 fellow eyes were correctly classified with the mixed index.

**Fig 8:**
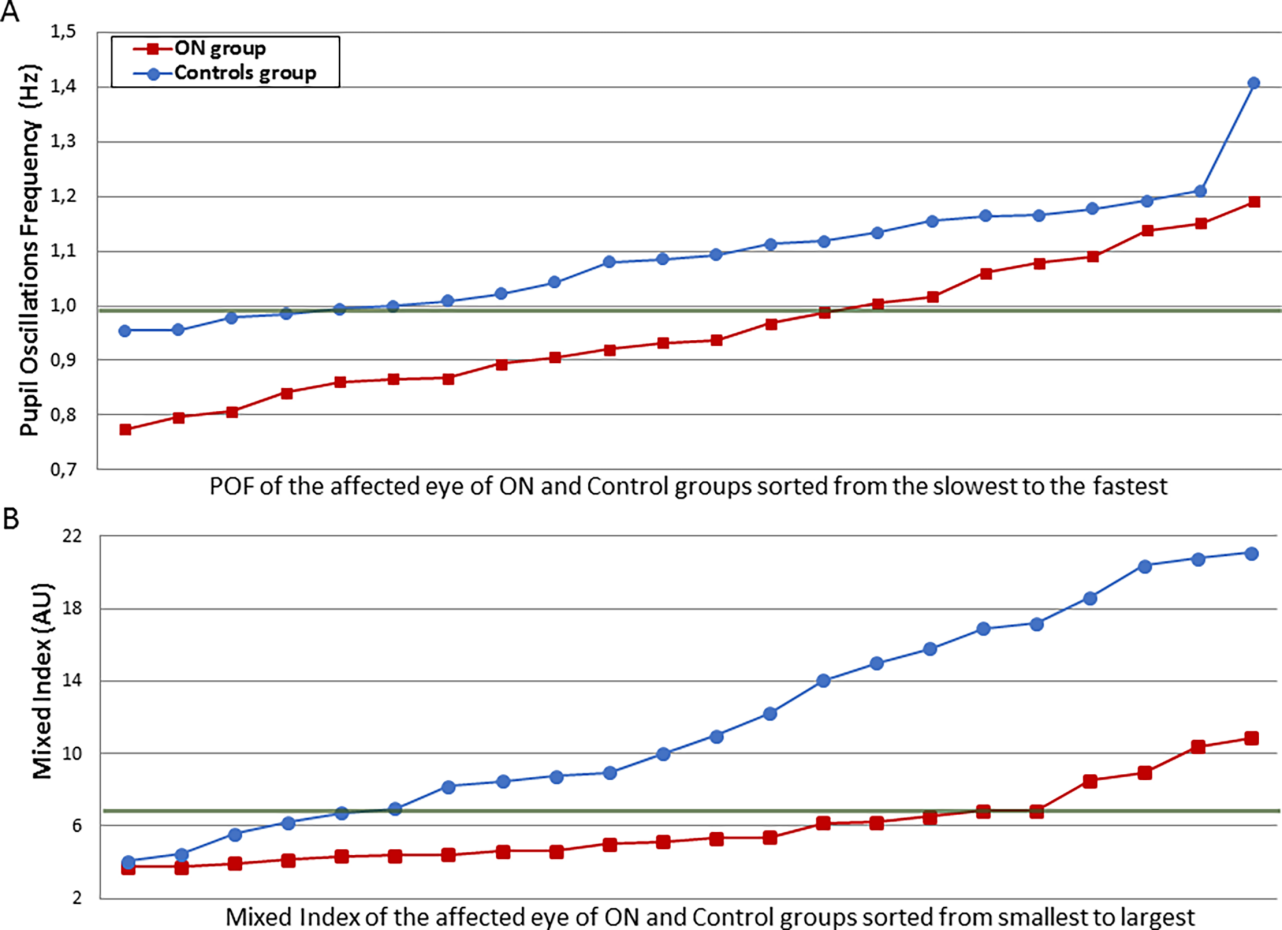
Individual results of the affected eye of the ON group and of the control participants. A. Individual results of the affected eye of ON patients and control participants sorted by increasing POF; the green line represents the threshold (0.99 Hz) that best discriminate the 2 groups. B. Individual results of the affected eye of ON patients and control participants sorted by increasing Mixed Index values. The green line represents the threshold (6.89 AU) that best discriminate the 2 groups.

We then applied the same method to compute ROC curves using the affected eye of patients and the homologous “affected” eye of Control participants (Fig 7). When using solely the POF, we found an Area Under the Curve of 0.75, a threshold of 0.99 Hz, a sensitivity of 64% (95%CI: 41%-83%) and a specificity of 82% (95%CI: 60%-95%). Of the 22 patients, 14 were correctly classified, and 18/22 control subjects were correctly classified. With a mixed (POF/SD) index, we obtained an AUC of 0.82 (Fig 7); with a threshold of 6.89 arbitrary units, the sensitivity was 82% (95%CI: 60%-95%) and the specificity was 77% (95%CI: 55%-92%), indicating that this mixed index was more powerful in identifying the affected eye of ON patients relative to controls. With this mixed index, 18/22 patients and 17/22 control subjects were correctly classified. The individual results sorted by increasing values are shown in Fig 8.

## Discussion

By coupling pupil size measured with infrared oculography to the luminance of a stimulus displayed on a standard computer screen, we could induce large and sustained pupil oscillations and test whether pupil oscillations were a reliable biomarker of Optic Neuritis. Using signal analyses to characterize the power and frequency of POs with a population of 22 patients diagnosed for unilateral ON within five years, and 22 matched control participants, we found that the frequency and variability of pupil oscillations allowed discriminating the affected and fellow eyes of patients, and patients from controls with a reasonable sensitivity and specificity. In contrast, the amplitude (or power) of POs was not a reliable index of the disease. We now discuss some of our findings in more details in the following.

### Binocular versus monocular POF

An intriguing finding is the differences of POF found in the monocular and binocular conditions. In all participants, the frequency of POs is higher in binocular than in monocular conditions. Moreover, in binocular conditions, the pupil data of patients is not significantly different from those of the control participants. This is surprising as one simple account of the fastest oscillations in binocular conditions, as compared to monocular conditions, is that the inputs from the two eyes are combined, which should increase the pupil drive and should speed-up the pupil response. Since the affected eye of patients shows slower POs, a simple summation process of the inputs from the two eyes should result in overall slower oscillations for patients than for control participants, which is not the case. This suggests that a simple summation of the inputs from the two eyes is not sufficient to account for the POF data in binocular conditions, and raises questions on how inputs from the 2 eyes are combined to drive the pupil.[3]

### Choice of the signal analysis method

The choice to analyze the pupil data over time (time-frequency analysis), rather than with direct FFT or other methods that are applied at once on the whole signal, permitted to compute the variability of the PO oscillations over time, which proved to be larger in the affected eye, as compared to the fellow eye, or to the eyes of control participant. As both the frequency and variability measures are independent and derived from a single recording, we computed a mixed index that does not rely on additional assumptions or data collection. This mixed index, based on both PO frequency and PO variability revealed greater sensitivity than PO frequency alone to discriminate patients from control participants.

### Usability of the method to routinely assess afferent pathway defects

It is well known that pupil reactivity to light is affected by drugs, such as benzodiazepines[22] or is perturbed in several, possibly concomitant pathologies, (e.g. cataract, autonomic neuropathy[23]). Drugs are not expected to have a major effect in the present study, because all patients had a history of *unilateral* Optic Neuritis. Thus, the effects of drugs should be similar for both eyes, and should not affect comparisons between the affected and fellow eyes. Although drugs can differently affect pupil reactivity across patients or between patients and controls, and thus limit comparisons of absolute pupil size, adjusting the response gain for each participant, and normalizing the pupil responses permits to overcome this issue. As a general rule, a strategy relying on *relative*, rather than *absolute*, comparisons of pupil dynamics, as was done here, should not suffer from this issue. In this view, it is worth noting that our method allows using *regional* stimuli presented in different location of the visual field, and to compare the *relative* POF obtained in different conditions in a single individual. On-going work indicates that, indeed, POs can be reliably induced with smaller stimuli than those used here.

In addition, as pupil activity is influenced by sympathetic inputs (see introduction), the characteristics of POs (mean pupil size, PO power and PO frequency) could be modulated by cognitive factors (attention or stress, for instance). We tested whether the attentional load modifies the PO characteristics with a group of 16 young healthy participants with visual tasks of varying difficulty (counting centrally presented targets within a sequence of distractors). The results showed that the attentional load modulated mean pupil size and mean PO power, but, importantly, not the PO frequency (in preparation). These results strengthen the view that POF is a more stable variable than PO amplitude, and is relatively immune to sympathetic modulation, although these preliminary findings need be extended to different populations.

### Further studies

Further studies are needed to improve the method, for instance to determine whether POF depends upon cognitive factors, such as attention, stress or fatigue. The method should also be tested with different pathologies (e.g. Retinitis Pigmentosa, Age Related Macular Disease, Glaucoma, etc.) to determine its sensitivity and specificity.

Moreover, whether the PO frequency depends on the Gain used to transform pupil data into luminance needs to be fully assessed as well as the effect of the stimulus color. This will be useful to determine whether the modulation of luminance can be decreased to avoid dazzling the participants or inducing photophobia, while still collecting reliable data. Preliminary tests with healthy subjects indicate that changing the Gain does not notably modify the POF, despite changes in the modulation power, indicating that the POF likely reflects the dynamics of pupil reactivity, which is specifically altered by the demyelination characteristic of ON. Moreover, repetition of POF measures in healthy subjects over several days indicated that the POF was stable over time.

## Conclusion

The present study confirmed that the retino-pupil loop reflects the dynamics of information transfer through the optic nerve, and its defects in neuropathies such as ON.[18–20] Although many studies probed ON and other pathologies using the PLR in response to a flash by analyzing the amplitude and latency of the pupil response, the present method relies on the measure of the frequency of PO, rather than the power or amplitude of the pupillary response. This requires recording pupil activity over 30–60 seconds, but presents the advantage that this measure is independent of the pupil modulation amplitude, which may be more contaminated by artifacts or specific settings than the oscillation frequency. An additional advantage is that this method can be used with a variety of low level stimulus characteristics, in particular with smaller stimuli displayed in specific regions of the visual field.

Overall, the present results indicate that coupling pupil size to stimulus luminance in real time provide robust data and can be employed with standard graphic displays. Although the present results only provide a proof of concept with a small group of patients with ON, this method will further be tested on population of controls and patients with different diseases, to determine whether POF is specific to ON, and to provide a reference data set against which individuals -and methods- can be compared.

Routinely assessing the POF of patients could bring a useful tool to characterize the dynamics of the retino-pupil loop whose functioning relies on the retinal neural circuitry and the optic nerve. Previous studies[18–20,22] already established the interest of characterizing pupil activity to assess the integrity–or defects- of the retinal neural circuits. The present study adds evidence in line with previous studies and describes a fast, simple, and reliable method and a signal analysis approach to assess the impact of optic neuropathies through pupil oscillatory behavior.

## Supporting information

S1 Fig Fundamental frequency of endogenous pupil oscillations computed with 3 different methods. Pupil Oscillation Frequency with maximum power in the 0.5–2 Hz range for the affected eye (triangles), the fellow eye (squares), and for binocular (circles) recordings, computed with three different methods: direct FFT, Auto-correlation and Time-Frequency maps for ON patients (dash lines) and for controls participants (continuous lines).
(TIF)

S1 Table Results of the ANOVA performed on the POF. Pupil oscillation frequency with maximum power in the 0.5-2Hz range computed with the time-frequency maps analysis.
(DOCX)

S2 Table Results of the ANOVA performed on POF variability. Variability of the POF was assessed as the standard deviation of the POF computed across time in the time-frequency maps analysis.
(DOCX)

S1 File Spreadsheets with the relevant data. (XLSX)
