# Supplementary figures and images for: A novel method of inducing endogenous pupil oscillations to detect patients with unilateral optic neuritis
Source: PLoS One. 2018 Aug 22;13(8):e0201730. doi: 10.1371/journal.pone.0201730 (PMC6104938; doi:10.1371/journal.pone.0201730)

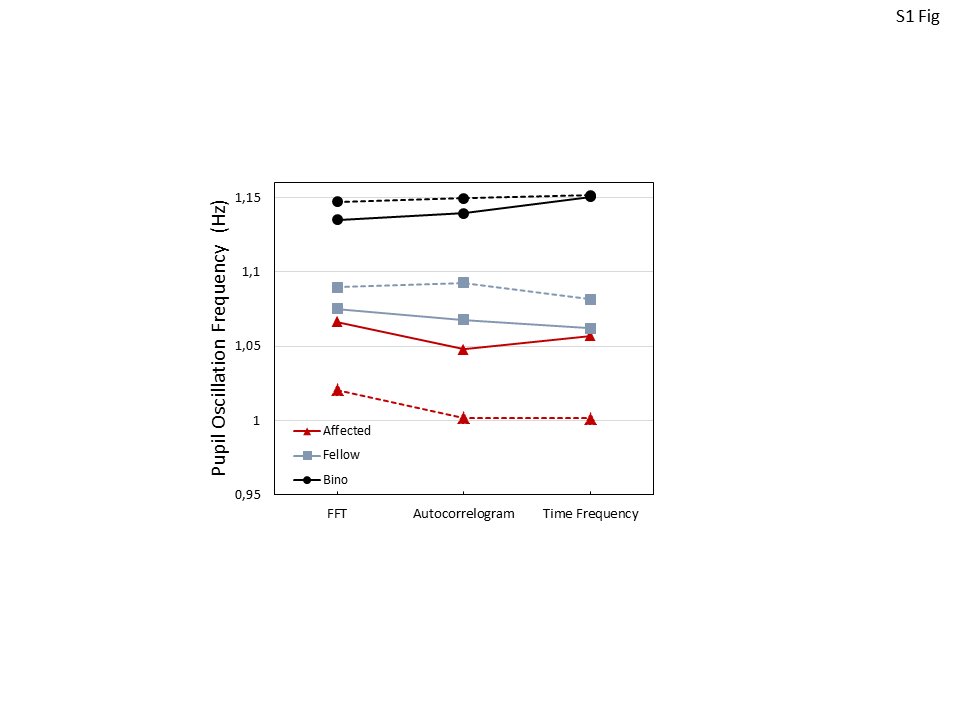

Supplement: S1 Fig — Pupil Oscillation Frequency with maximum power in the 0.5–2 Hz range for the affected eye (triangles), the fellow eye (squares), and for binocular (circles) recordings, computed with three different methods: direct FFT, Auto-correlation and Time-Frequency maps for ON patients (dash lines) and for controls participants (continuous lines). (TIF) [file pone.0201730.s001.tif]
