# Supplementary material for: A novel method of inducing endogenous pupil oscillations to detect patients with unilateral optic neuritis
Source: PLoS One. 2018 Aug 22;13(8):e0201730. doi: 10.1371/journal.pone.0201730 (PMC6104938; doi:10.1371/journal.pone.0201730)
Supplement: S1 Table — Pupil oscillation frequency with maximum power in the 0.5-2Hz range computed with the time-frequency maps analysis. (DOCX) [file pone.0201730.s002.docx]

**S1 Table**. **Results of the ANOVA performed on the POF.** Pupil oscillation frequency with maximum power in the 0.5-2Hz range computed with the time-frequency maps analysis.

|  | F | p | partial ᶯ² |
| --- | --- | --- | --- |
| **factor Group:**  **optic neuritis, controls** | **(1, 242)=9.53** | **2_*_10^-3^** | **0.04** |
| **factor Eye:**  **affected, fellow, binocular** | **(2, 242)=29.01** | **<10^-3^** | **0.19** |
| **interaction between factors**  **Group and Eye** | **(2, 242)=7.12** | **<10^-3^** | **0.06** |
