# Supplementary material for: A novel method of inducing endogenous pupil oscillations to detect patients with unilateral optic neuritis
Source: PLoS One. 2018 Aug 22;13(8):e0201730. doi: 10.1371/journal.pone.0201730 (PMC6104938; doi:10.1371/journal.pone.0201730)
Supplement: S2 Table — Variability of the POF was assessed as the standard deviation of the POF computed across time in the time-frequency maps analysis. (DOCX) [file pone.0201730.s003.docx]

**S2 Table. Results of the ANOVA performed on POF variability.** Variability of the POF was assessed as the standard deviation of the POF computed across time in the time-frequency maps analysis.

|  | F | p | partial ᶯ² |
| --- | --- | --- | --- |
| **factor Group:**  **optic neuritis, controls** | **(1, 242)=26.50** | **<10^-5^** | **0.10** |
| **factor Eye:**  **affected, fellow, binocular** | **(2, 242)=15.66** | **<10^-5^** | **0.11** |
| interaction between factors  Group and Eye | (2, 242)=1.00 | 0.37 | 0.01 |
